# BRD7在非小细胞肺癌组织中的表达及临床意义

**DOI:** 10.3779/j.issn.1009-3419.2011.10.11

**Published:** 2011-10-20

**Authors:** 龙 陈, 风雷 喻

**Affiliations:** 410000 长沙，中南大学湘雅二医院心胸外科 Department of Cardiothoracic Surgery, Second Xiangya Hospital, Central South University, Changsha 410000, China

**Keywords:** 肺肿瘤, 溴区包含蛋白7, 免疫组化, Lung neoplasms, Bromodomain-containing protein 7, Immunohistochemistry

## Abstract

**背景与目的:**

溴区包含蛋白7（bromodomain-containing protein 7, BRD7）基因属于bromodomain家族成员，该家族的大多数成员与上皮类肿瘤的发生密切相关。本研究旨在探讨BRD7在非小细胞肺癌（non-small cell lung cancer, NSCLC）中的表达及其临床意义。

**方法:**

采用免疫组化法检测101例NSCLC癌组织及其33例正常肺组织中BRD7的表达情况。

**结果:**

BRD7在NSCLC组织中的阳性表达率明显高于正常肺组织；BRD7在淋巴结转移组阳性表达率高于无淋巴结转移组；BRD7的阳性表达率随着TNM分期的升高而升高。BRD7的阳性表达与患者年龄、性别、吸烟情况、病理类型、分化程度无关（*P*>0.05）。

**结论:**

BRD7在NSCLC中有较高的表达率，除了与淋巴结转移有关外，还与TNM分期有关，提示BRD7可能与肺癌的发生、发展和转移有关。

## Abstract (en)

**Background and objective:**

The bromodomain-containing protein 7 (BRD7) gene belongs to the bromodomain family. The majority of the members of this family are closely related to epithelial tumors. The aim of this study is to explore BRD7 expression and its clinical significance in non-small cell lung cancer (NSCLC).

**Methods:**

BRD7 protein expression was detected in tissues samples from 101 NSCLC cases and 33 normal lung tissue samples using streptavidin-peroxidase immunohistochemistry.

**Results:**

BRD7 expression was significantly higher in cancer tissues than that in normal lung tissues. Positive BRD7 expression in the lymph node metastasis group was significantly higher than that in the non-lymph node metastasis group. The positive expression rate of BRD7 increased with the increasing Tumor-Nodes-Metastasis (TNM) stage. No significant differences in positive BRD7 expression were observed with age, smoking, gender, pathology type, and degree of differentiation among the NSCLC patients (*P*>0.05).

**Conclusion:**

BRD7 is highly expressed in NSCLC. In addition to the degree of differentiation and extent of lymphatic metastasis, BRD7 expression is correlated with TNM stage, which indicates that BRD7 may be related to the occurrence, development, and metastasis of lung cancers.

溴区包含蛋白7（bromodomain-containing protein 7, *BRD7*）基因属于bromodomain家族成员，该家族的大多数成员与上皮类肿瘤、恶性血液病的发生密切相关^[1]^。过表达*BRD7*基因可抑制鼻咽癌细胞增殖和细胞周期进程，并部分逆转鼻咽癌细胞的恶性表型^[2]^。基于BRD7在鼻咽癌中的作用及与上皮类肿瘤的密切关系，推测BRD7可能参与肺癌的发生和发展。本研究旨在通过免疫组化技术检测BRD7在非小细胞肺癌（non-small cell lung cancer, NSCLC）组织中的表达，探讨*BRD7*基因在NSCLC临床和病理诊断中可能的作用及意义。

## 1 材料与方法

### 1.1 一般资料

收集2009年1月-2011年5月中南大学湘雅二医院有明确病理诊断的NSCLC手术标本101例作为实验组，并取其中33例正常组织（距癌组织>10 cm）标本作为对照组，以上标本切取后立即置于液氮中保存备用，其余部分常规送病理检查。

101例NSCLC包括男性81例，女性20例，年龄36岁-78岁，平均53岁。根据2004年WHO肺癌分类标准进行组织学分类：鳞癌44例，腺癌52例，大细胞癌5例；高分化7例，中分化73例，低分化16例（大细胞癌属于未分化癌，故未纳入该分组）。依据国际抗癌联盟2009年修订的肺癌TNM分期标准进行分期：Ⅰ期52例，Ⅱ期17例，Ⅲ期32例；伴有淋巴结转移的患者47例，无淋巴结转移的患者54例。所有患者术前均未行化疗、放疗等辅助治疗。

### 1.2 实验试剂与方法

大鼠抗-BRD7单抗原液（1.0 mg/mL）购自美国Sigma生物技术公司，即用型二步法大鼠免疫组化检测试剂盒、DAB显色试剂盒均购自北京中杉金桥生物技术有限公司。

试验方法：标本经4%多聚甲醛固定，常规脱水包埋，4 μm厚连续切片，石蜡切片常规HE染色进行组织学认定后，采用免疫组化二步法检测BRD7在不同肺组织中的表达。DAB显色，以PBS代替一抗作阴性对照，用已知阳性肺癌切片作阳性对照。

### 1.3 实验结果及判断标准

细胞内呈棕色颗粒者为阳性染色。根据染色细胞百分率和染色程度进行评定和分析。每例均随机观察10个高倍视野（×400），每个视野计数100个肿瘤细胞，阳性细胞率计分标准：≤10%为0分，11%-25%为1分，26%-50%为2分，51%-75%为3分，>75%为4分。染色强度：浅棕色为1分，棕色为2分，深棕色为3分。最后按乘积分数分为4个等级：0分为阴性（-），1分-4分为弱阳性（+），5分-8分为中度阳性（++），9分-12分为强阳性（+++）。结果判断：将（-）和（+）定为阴性表达，（++）和（+++）定为阳性表达。

### 1.4 统计学处理

应用SPSS 13.0统计软件，组间比较采用卡方检验。*P* < 0.05为差异有统计学意义。

## 2 结果

### 2.1 BRD7在NSCLC癌组织、正常肺组织中的表达

BRD7蛋白的表达以细胞核为主，在正常肺组织中很少见到浅棕色颗粒，表明BRD7蛋白在正常肺组织中低表达或不表达；在癌组织中可以看到大部分组织都出现较多的棕色或深棕色颗粒，显示BRD7蛋白表达明显增强（图 1）。BRD7蛋白在正常肺组织组及NSCLC组表达阳性率分别为6.1%（2/33）和76.2%（77/101），两组相比差异有统计学意义（*χ*^2^=50.622, *P* < 0.001）。

**1:**
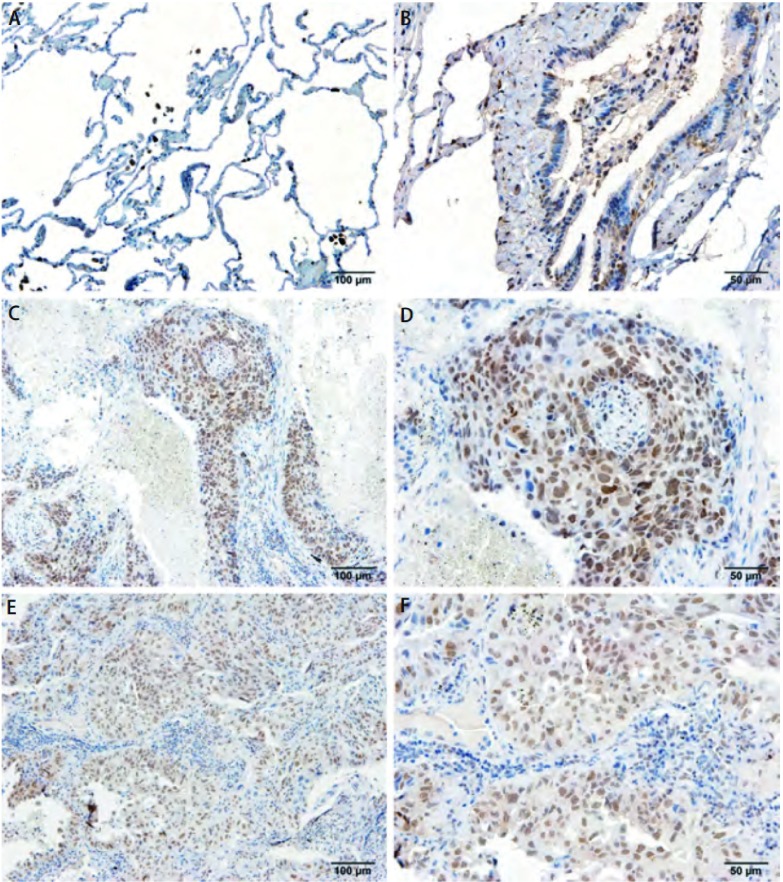
BRD7在不同组织中的表达情况（SP法；A、C、E×100；B、D、F×400）。A、B：BRD7在正常肺组织中低表达；C、D：在鳞癌中高表达；E、F：在腺癌中高表达 The expression of BRD7 in different tissues (SP method; A, C, E×100; B, D, F×400). A and B: low expression of BRD7 in normal lung tissue; C and D: high expression in squamous cell carcinoma tissue; E and F: high expression in adenocarcinoma tissue

### 2.2 BRD7的表达与NSCLC临床病理特征的关系

有淋巴结转移的患者BRD7的阳性表达率（89.4%）高于无淋巴结转移的患者（64.8%），差异有统计学意义（*P*=0.004）。BRD7的阳性表达率随着TNM分期的升高而升高，Ⅰ期、Ⅱ期、Ⅲ期患者中分别为65.4%、76.5%、93.8%，差异有统计学意义（*P*=0.012）。BRD7的表达与患者性别、年龄、吸烟、病理类型、分化程度无关（*P*>0.05）（表 1）。

**1:** BRD7的表达水平与非小细胞肺癌临床病理特征的关系 Relationship between BRD7 expression and clinicopathological characteristics of non-small cell lung cancer

Varial/Category	Entire sample (*n*=101)	BRD7 expression		Positive ratio (%)	*χ*^2^	*P*
		(-)	(+)			
Age (yr)					0.193	0.660
≥55	75	17	58	77.3		
< 55	26	7	19	73.1		
Gender					0.536	0.464
Male	81	18	63	77.8		
Female	20	6	14	70.0		
Smoking history					0.403	0.526
Ever	52	11	41	78.8		
Never	49	13	36	73.5		
Histopathology					1.771	0.413
SCC	44	8	36	81.8		
ADC	52	14	38	73.1		
LCC	5	2	3	60.0		
Differentiation^*^					5.666	0.059
Well	7	4	3	42.9		
Moderate	73	16	57	78.1		
Poor	16	2	14	87.5		
P-TNM status					8.799	0.012
Ⅰ	52	18	34	65.4		
Ⅱ	17	4	13	76.5		
Ⅲ	32	2	30	93.8		
Lymph node					8.358	0.004
N0	54	19	35	64.8		
N1-3	47	5	42	89.4		
SCC: squamous cell carcinoma; ADC: adenocarcinoma; LCC: large cell carcinoma; ^*^This group is not included in the large cell carcinoma (*n*=96).						

## 3 讨论

肺癌是全球发病率和死亡率最高的恶性肿瘤，其中约有80%为NSCLC。近年来NSCLC发病率在世界范围内呈上升和年轻化趋势，其发生、发展和转移是一个极其复杂的多基因调控异常的过程^[3, 4]^。因此，研究肺癌的病因、发病机制、寻找新的治疗靶点具有重要的临床意义。*BRD7*基因是1999年克隆的一个新的bromodomain基因，该基因定位于染色体16q12.1-12.2，cDNA全长2, 317 bp，其编码产物BRD7蛋白是一种含溴区（bromodomain）结构域的蛋白质，属于bromodomain家族成员^[5]^。溴区结构域是一个进化上高度保守的功能结构域，可特异性地与组蛋白末端乙酰化的赖氨酸位点结合，并将核内的组蛋白乙酰化信号传递给转录相关的蛋白质复合物，通过改变染色质的构象参与基因转录调控^[6]^。近年来国内研究人员对BRD7基因在鼻咽癌中的功能研究结果^[7, 8]^表明，BRD7在鼻咽癌中具有抑瘤作用。

研究^[9-11]^表明*BRD7*基因是一个p53依赖的抑癌基因，*BRD7*基因与p300相互作用，通过溴区结构域连接乙酰化组蛋白，帮助维持p53转录时连接位点的组蛋白在一个合适的乙酰化状态；同时*BRD7*基因被鉴别为BRG1的特有的染色体重塑复合体，可减弱p21的表达，从而降低p53和p21启动子连接，促进p53导致核小体重新分布而发生染色体重塑。此外*BRD7*基因作为一个其它蛋白转录辅助因子参与Ras/MEK/ERK和Rb/E2F途径的调节^[1]^。因此BRD7不仅参与p53调控，也参与细胞周期管理基因的调节。

本研究对NSCLC的病理标本进行免疫组化检测，首次方便、快速、高效地检测了BRD7在NSCLC中的表达，并探讨其与肺癌患者性别、年龄、吸烟情况、组织类型、分化程度和淋巴结转移情况以及临床分期的关系，从而进一步探讨BRD7在肺癌中的作用。本研究发现BRD7在NSCLC癌组织中有较高阳性表达率，说明BRD7在NSCLC中的表达具有较高的敏感性。通过进一步分析发现BRD7在NSCLC中的表达与性别、年龄、吸烟情况、组织类型及分化程度无关，提示BRD7靶点的NSCLC治疗有较广泛的适宜人群。BRD7在有淋巴结转移组的阳性表达率高于无淋巴结转移组，不同TNM分期中BRD7的阳性表达率随着分期的升高而升高，提示BRD7可以作为针对促进肿瘤转移的在体标志物。许多研究^[7-10]^表明，BRD7可促进细胞的运动、粘附及迁移，在细胞生长、生存、转化中也具有重要作用，提示BRD7可能与肺癌的发生、发展密切相关。

研究^[12]^发现人类肿瘤的半数都发生p53的突变，且突变多发生在外显子5-8。当*p53*基因发生突变时其介导的细胞周期调节失控，DNA合成程序紊乱，发生遗传不稳定性及多倍性，也可能使p53介导的凋亡丧失而导致肿瘤的发生。突变的*p53*基因不仅抑癌活性丧失，而且具备促进癌症发生的作用，*p53*基因由抑癌基因转变成癌基因。*p53*基因突变是肺癌中发生频率最高的遗传改变。Mechanic等^[13]^研究表明*p53*基因突变谱的不同可能影响到肺癌的易感性以及相应肿瘤形成的结论。*BRD7*与*p53*有交互作用，被认为是肿瘤抑制基因。BRD7在NSCLC中出现高表达，可能是由于*BRD7*基因突变，而使BRD7正常的蛋白功能丧失，因此虽然在肺癌细胞中呈现高表达的趋势，但是实际已经丧失了抑制肿瘤功能，这种表达模式与p53类似。

BRD7与NSCLC的发生和发展有密切的关系，深入研究*BRD7*基因对于了解肺癌的发生、发展具有重要意义。BRD7在肺癌细胞中是否存在突变以及存在哪些位点的突变，尚需要进一步的深入研究。
